# Performance, thermoregulation, and liver function in beef heifers exposed to endophyte-infected or endophyte-free tall fescue under a common environment

**DOI:** 10.1093/tas/txaf171

**Published:** 2026-01-09

**Authors:** Joao Vitor G Takashe, Jackson Matthews, Daniel W Shike, Camila U Braz

**Affiliations:** Department of Animal Sciences, University of Illinois at Urbana-Champaign, Urbana, IL, 61801, United States; Department of Animal Sciences, University of Illinois at Urbana-Champaign, Urbana, IL, 61801, United States; Department of Animal Sciences, University of Illinois at Urbana-Champaign, Urbana, IL, 61801, United States; Department of Animal Sciences, University of Illinois at Urbana-Champaign, Urbana, IL, 61801, United States

**Keywords:** beef cattle, ergot alkaloids, feed intake, fescue toxicosis, liver function

## Abstract

Fescue toxicosis, induced by ingestion of ergot alkaloids from endophyte-infected tall fescue, remains a significant challenge to beef cattle production. This study evaluated the effects of fescue toxicosis on performance, thermoregulation, and liver metabolism in beef heifers maintained under the same experimental conditions as non-exposed controls. Twenty-four commercial Angus heifers were randomly assigned to a diet containing either an endophyte-infected (E+) or endophyte-free (E−) seeds for 49 days. Heifers were allocated to balance genetic representation across the two dietary groups to minimize genetic confounding. Environmental data, including temperature and relative humidity, were continuously recorded, and minimum, mean, and maximum temperature-humidity index (THI) were calculated each day. Two heatwave events occurred during the trial. E + heifers exhibited reductions in dry matter intake (DMI), feeding rate, meal size, average daily gain (ADG), and body weight, and elevated respiration rate and rectal temperature compared to E− heifers (*P <*0.05). Physiological impairments emerged within the first two weeks of exposure, with a subsequent heatwave exacerbating stress in the E + group. Among the three THI metrics evaluated, minimum THI showed the strongest negative correlation with DMI (*r = *−0.20). The two heatwave events coincided with notable reductions in DMI across both dietary groups. However, DMI was more severely suppressed in E + heifers compared to the E− group. Serum analysis revealed elevated aspartate aminotransferase (AST) and reduced alkaline phosphatase (ALP) and cholesterol levels in E + heifers, indicating liver stress and metabolic dysfunction (*P <*0.05). No significant differences were observed in serum albumin, bilirubin, gamma-glutamyltransferase (GGT), or triglyceride levels between groups. Collectively, these findings underscore the complex metabolic and physiological disturbances triggered by ergot alkaloid exposure, which compromise heifer health, thermoregulation, and productivity, particularly under heat stress conditions.

## Teaser

This study demonstrates that toxins in endophyte-infected tall fescue reduce feed intake, growth, and heat tolerance in beef heifers, with these effects becoming apparent within two weeks. The exposure also caused liver stress and metabolic disruption. These findings highlight the significant impacts of fescue toxicosis on cattle health and productivity.

## Introduction

Tall fescue is a cool-season perennial grass and the predominant forage used in beef cattle operations across the Southeastern United States, covering nearly 14 million hectares and used as pasture, silage, and hay (Kallenbach 2015). Its popularity stems from several agronomic advantages, including ease of cultivation, high durability, stockpiling potential, and minimal maintenance requirements (Ferguson et al. 2021). Additionally, tall fescue is valued for its extended grazing season, resilience under heavy grazing pressure, scalability, and resistance to drought and pests, attributed to its symbiotic relationship with the endophytic fungus *Epichloë coenophiala* (Young et al. 2013). Tall fescue is expected to become increasingly important forage in livestock systems as temperatures rise due to climate change (Young et al. 2013; McCulley et al. 2014). However, despite these benefits, the presence of endophytic fungi in tall fescue results in the production of alkaloid compounds, such as ergovaline that, when consumed by cattle, sheep, or horses, induces a condition known as fescue toxicosis (Rhodes et al. 1991; Nihsen et al. 2004; Shoup et al. 2016).

Fescue toxicosis has detrimental effects on animal health and productivity. This condition is primarily characterized by vasoconstriction (the narrowing of blood vessels), which increases pulmonary arterial pressure and reduces blood flow (Rhodes et al. 1991). These circulatory impairments limit heat dissipation, leading to elevated body temperature and increased respiration rates (Eisemann et al. 2014; Shoup et al. 2016). Additionally, fescue toxicosis disrupts the endocrine system, further compounding its negative effects on overall physiologi­cal function (Burke et al. 2001; Burke and Rorie 2002). As a result, affected cattle experience decreased body weight, reduced feed intake, impaired reproduction efficiency, and lower milk production (Jackson et al. 1984; Schmidt et al. 1986; Burke and Rorie, 2002; Strickland et al. 2009). The cumulative effect of these outcomes contributes to a substantial economic burden, with fescue toxicosis accounting for approximately $2 billion in annual losses to the U.S. beef industry (Kallenbach 2015).

Several strategies have been proposed to mitigate the negative effects of fescue toxicosis, including both pasture- and animal-based solutions (Kallenbach 2015; Melchior and Myer 2018; Ferguson et al. 2021; Alfaro et al. 2023; Valente et al. 2024). These approaches focus on developing non- or low-toxic tall fescue varieties and supplementing cattle with vitamins, minerals, proteins, and administration of dopamine precursor. Pasture conversion is a straightforward and common procedure and can be successful when the seed transfer is minimized. However, pasture may view pasture renovation as a risky practice due to the high failure rate of pasture establishment and the gradual encroachment of toxic tall fescue over time ­(Kallenbach 2015; Ferguson et al. 2021). Additionally, supplementation strategies have shown limited success in mitigating fescue toxicosis symptoms (Melchior and Myer 2018; Alfaro et al. 2023).

The cow–calf sector is particularly affected by fescue toxicosis, as cows remain on toxic pastures throughout their productive lifespan while generating calves that become herd replacements or enter the meat production system (Kallenbach 2015; Ferguson et al. 2021). Studies focusing on beef heifer’s response to fescue toxicosis are scarce in the literature Therefore, this study aimed to compare the performance, thermoregulation, and liver function of beef heifers consuming endophyte-infected or endophyte-free tall fescue under identical environmental conditions.

## Materials and methods

### Experimental design

The study protocol was approved by the Institutional Animal Care and Use Committee (IACUC) at the University of Illinois at Urbana-Champaign (#23073). A total of 24 commercial Angus heifers were evaluated over a 70-day period (June 14^th^ to August 21^st^, 2023) in a feedlot-based system at the University of Illinois Beef and Sheep Field Research Laboratory (Urbana, IL). Heifers had no prior exposure to an endophyte-infected diet before the experiment. At the start of the trial, heifers had an average body weight of 367.6 kg (± 34 kg) and an average age of 295.3 days (± 4.3 days). To investigate the effects of endophyte-infected tall fescue (E+) consumption, heifers were assigned to two dietary groups (*n* = 12/group) and housed in four pens (*n* = 6/pen). The treatment group received E + tall fescue seeds, while the control group was fed endophyte-free (E−) tall fescue seeds as part of the total mixed ration. Both groups were managed under identical conditions, including housing in the same barn, same diet composition (except for the seeds), feeding system, and water availability. The barn was fully enclosed with a gable roof and featured slatted concrete floors covered with rubber matting positioned above shallow gravity-flow flush pits. It contained 20 pens, each approximately 5 x 5 meters in size, and each equipped with a single Vytelle feed bunk. Heifers were carefully allocated to balance genetic representation across the two dietary groups. Specifically, each group included progeny from four different sires with equal representation. All heifers were offspring of first-calf dams (Table S1).

Following the Beef Improvement Federation (BIF, 2016) guidelines, heifers underwent a 21-day adaptation period to the Vytelle GrowSafe System prior to the experiment to eliminate potential bias in feeding behavior. This system records real-time individual feed consumption intake and patterns, enabling a detailed analysis of each animal’s feeding behavior and intake dynamics. Each heifer was equipped with an electronic identification ear tag (AllFlex, Inc., Dallas, TX, USA) to track feed bunk visits and feeding activity.

Fescue seed quantities were determined based on an ergovaline dosage of 20 μg/kg of body weight per day, the threshold required to induce characteristic signs of fescue toxicosis in beef cattle (Holtcamp et al. 2019; Alfaro et al. 2021). The ergovaline concentration in the E + seeds (6.76 µg/g) was obtained via high-performance liquid chromatography (HPLC) analysis conducted by the Veterinary Medical Diagnostic Laboratory at the University of Missouri (Columbia, MO 65211). Heifers in the E− group received an equal quantity of seeds as those in the E + group. The total seed allotment was adjusted weekly and incorporated into the diet for 49 days to maintain a consistent ergovaline dosage delivery on body weight basis. The diet was formulated to meet the nutrient requirements (Table 1) according to the National Research Council (NRC) guidelines and was also adjusted weekly to accommodate the heifers’ changing nutritional needs.

**Table 1.** Diet composition fed to the heifers during the experiment, presented as dry matter (DM) basis.

Ingredient	% DM	% of ration, DM
**Tall fescue seeds^a^**	90	12
**Corn silage**	30	22
**Modified distillers grains**	48	22
**High moisture corn**	60	9
**Dry rolled corn**	85	26
**Supplement^b^**	90	9
Nutrient Composition		
** Crude protein**	8.84	
** Neutral detergent fiber**	27.85	
** Acid detergent fiber**	13.31	

a The estimated amount of seed provided per heifer at the beginning and the end of the trial was 1.02 kg, and 1.34 kg (as fed), respectively.

b 76.2% Ground yellow corn, 15.89% Limestone, 6% Urea, 0.91% Dairy trace minerals, 0.16% Rumensin 90, 0.1% Tylan 40, 0.75% Choice white Grease.

### Measurements and sampling

Throughout the experiment, the effect of fescue toxicosis on performance, body weight, feed intake, and feeding behavior was monitored and evaluated. Body weight was recorded once per week using a digital scale, consistently in the early morning prior to feeding time (0700 h). Feed intake and feeding behavior were continuously assessed using an electronic feed monitoring system (Vytelle GrowSafe^®^ Systems Ltd, Airdrie, Alberta, ­Canada), as described by Sowell et al. (1998). Feeding behavior traits included feeding frequency, feeding duration, feeding rate, meal frequency, meal duration, and meal intake (meal size). Feed intake was expressed as dry matter intake (DMI), defined as the average daily amount of feed consumed on a dry matter basis (kg/d) over a period of at least 35 days with reliable feed intake measurements. DMI provides a more accurate estimate of actual nutrient intake, as water content in feeds can vary significantly (McGee et al. 2014). Feeding frequency was defined as the number of daily visits to the feed bunk (visits/d). Feeding duration represented the total daily time spent eating (min/d). Feeding rate was calculated as the amount of feed consumed per unit of time (g/min). Meal frequency quantified the number of distinct meals consumed daily (meal/d). Meal duration indicated the average time spent consuming each meal (min/meal). Meal intake represented the amount consumed per meal (g/meal). Meals were defined using a standardized meal criterion interval of 5 min (300 seconds) between feeding events (Bailey et al. 2012).

To evaluate the interactive effects of ambient temperature and fescue toxin exposure on daily feed intake, environmental data, including temperature and relative humidity were continuously recorded inside the barn using a SwitchBot IP65 Wireless Indoor/Outdoor Hygrometer-Thermometer. The sensor was positioned at the center of the experimental site, between the treatment and the control groups, aligned with the feed bunks, and approximately 2.5 m above the floor. This device measured temperature (T_C_) with an accuracy of ±0.2°C and relative humidity (RH) with an accuracy of ±1.8% RH, sampling data every 4 seconds. The temperature-humidity index (THI) was calculated as: $\mathrm{THI}=T_{C}-(0.55-0.0055\times RH)\;(T_{C}-14.5)$, where $T_{C}$ represents ambient temperature (°C) and *RH* represents relative humidity (%). THI is commonly used to indicate the degree of stress on cattle. To determine which THI metric best explained the variation in feed intake observed during the trial, we evaluated the correlation between daily feed intake and the minimum, mean, and maximum THI values recorded each day. Environmental data, including temperature, relative humidity, and THI throughout the experimental period are displayed in Fig. S1.

The impact of fescue toxicosis on heifer thermoregulation was evaluated weekly by measuring rectal temperature and respiration rate. Rectal temperature was recorded using a digital rectal thermometer (Sharptemp V, Cotran Corporation, Portsmouth, RI, USA) while heifers were restrained in the chute early in the morning (0700 h). The collection order was consistent throughout the experiment, proceeding sequentially from pen 1 to pen 4. Respiration rate was determined by timing 20 uninterrupted flank movements using a stopwatch, with each inhalation and exhalation counted as one breath. The total count was then converted to breaths per minute. Liver metabolism was also assessed at the end of the trial by evaluating serum liver profile biomarkers in the heifers. For this purpose, blood samples were collected via jugular venipuncture using 20-gauge needles and transferred into the serum collection tubes (BD Vacutainer, Franklin Lakes, NJ, USA). Samples were left at room temperature for 30 minutes to allow clot formation before centrifugation at 1300x g for 20 min at 5°C. The resulting serum was carefully transferred into new tubes for subsequent analyses. Refrigerated samples were sent to the UIUC Veterinary Diagnostic Laboratory for liver profile analysis. The liver profile included measurements of albumin (ALB), alkaline Phosphatase (ALP), aspartate aminotransferase (AST), gamma-glutamyl transferase (GGT), total bilirubin, total cholesterol, glutamate dehydrogenase (GLDH), and triglycerides (Table S2).

### Statistical analysis

**Fig. 1.**
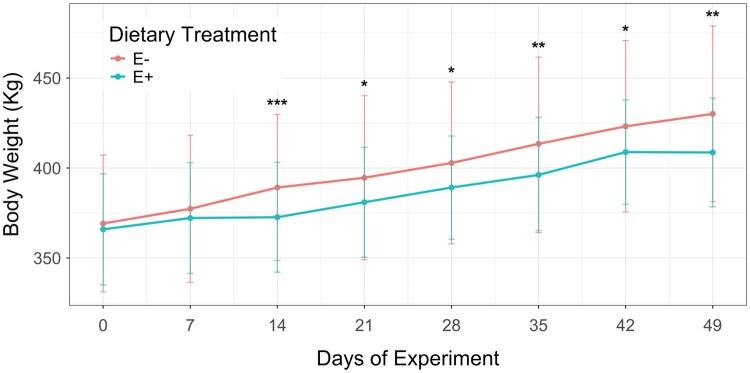
Body weight of heifers fed endophyte-infected (E+) or endophyte-free (E−) tall fescue seeds over a 49-day experimental period. Body weight was recorded weekly. Vertical bars represent the standard deviation for each dietary treatment. Asterisks indicate statistical significance between treatments at each time point: *P ≤0.05; **P ≤0.01; ***P ≤0.001. Figure was generated using R package "ggplot2" (https://cran.r-project.org/web/packages/ggplot2/).

The effects of E + consumption on body weight, DMI, rectal temperature, and respiration rate traits were evaluated using a linear model with the *lm* function in R, including dietary treatment (E + or E−) as a fixed effect. Initial body weight (${BW}_{0}$, day 0, Fig. 1) was used as a covariate in the analysis of body weight across the trial. For the analysis of the weekly average daily gain (ADG), the corresponding weekly average DMI was included in the model as a covariate to determine whether the effect of E + ingestion on ADG was primarily driven by differences in feed intake. Mid-test metabolic weight (${BW}_{0}+[\left({BW}_{7}-{BW}_{0}\right)/2]$, where ${BW}_{7}$ is the final body weight) was included as a covariate in the average DMI analysis (Pires et al. 2022). Feeding duration, feeding rate, meal intake, and meal duration were analyzed as repeated measures using the *lme* function from the *lmerTest* R package (Kuznetsova et al. 2017), with dietary treatment included as a fixed effect, animal as a random effect, and an appropriate covariance structure to account for repeated observations. Feeding frequency and meal frequency were analyzed using generalized linear mixed models with a Poisson distribution, implemented via *glmer* function in the *lme4* R package (Bates et al. 2015). In these models, dietary treatment was included as a fixed effect, while the animal was considered a random effect. Liver profile traits were analyzed using a linear model that included dietary treatment as a fixed effect and the average DMI from the five days prior to blood collection as a covariate to account for variation in feed intake. All data were reported as least squares means (± SEM). For count traits, means were presented as exponentiated least squares means for each group. Pen was not included as a random effect in the statistical model because the assignment of heifers to the pen was random, and the design was balanced, with an equal number of pens per treatment and an equal number of heifers per pen. Under these conditions, the treatment least squares means are considered unbiased (St-Pierre, 2007). Statistical significance was achieved with a *P*-value ≤0.05 and trends at *P*-value >0.05 to *P*-value ≤0.10.

## Results and discussion

Fescue toxicosis remains one of the most economically significant health challenges in the beef cattle industry. Despite decades of research, the full extent of ergot alkaloid toxicity in cattle is not yet fully understood. Previous studies have reported inconsistent findings. For instance, reductions in body weight associated with ergovaline exposure were observed by Brown et al. (2009) and Jackson et al. (2015), but not by Shoup et al. (2016). Similarly, Shoup et al. (2016) reported increased respiration rates in E + group, whereas Rahimabadi et al. (2022) did not. In addition, Brown et al. (2009) and Rice et al. (1997) found reductions in both ALP and AST levels in E + cattle, while Rahimabadi et al. (2022) reported elevated AST, and Jackson et al. (2015) observed no significant effect of the E + tall fescue ingestion on ALP or AST activity. Such discrepancies likely reflect differences in genetic background, environmental conditions, and concentration and composition of ergot alkaloids among the groups studied, or population-specific responses. To compare E + and E− animals under the same environmental conditions, we conducted a 49-day feeding trial to evaluate the effects of fescue toxicosis in beef heifers. Animals were assigned to one of two dietary treatments: a control group receiving endophyte-free (E−) tall fescue seed and a treatment group receiving endophyte-infected (E+) seed, with both groups receiving diets formulated to be nutritionally equivalent, representing similar genetic backgrounds, and maintained under the same environment. We assessed the impact of ergot alkaloid exposure on body weight, feed intake, feeding behavior, rectal temperature, respiration rate, and liver function, offering insights into the physiological and behavioral responses of beef cattle to fescue toxicosis.

### Impact of toxic fescue on heifers’ performance

Effects of the fescue toxin exposure on body weight became evident after two weeks of ingestion (*P <*0.05), as indicated by the reduced weight gain among E + heifers between days 7 and 14 (0.064 kg/day; Fig. 1, Table S3). During the same period, E− heifers gained approximately 11 kg on average (1.686 kg/day; Fig. 1, Table S3). Following this initial decline, E + heifers appeared to partially compensate for the reduced growth, with an ADG of 1.19 kg, significantly different from the ADG of E− heifers when accounting for feed intake variation (*P = *0.014). Thereafter, weekly body weight gains of E + heifers were comparable to those of E− heifers, averaging around 10 kg per week throughout the trial. However, E + heifers never reached the same overall body weight as E− heifers (Fig. 1). In the final week of the trial (days 42 to 49), the average body weight of E + heifers slightly decreased from 411 kg to 410 kg (−0.032 kg/day), whereas E− heifers gained approximately 7 kg (0.989 kg/day) during the same period. According to these findings, ergot alkaloids exposure affected weight gain during the second week of ingestion; after this period, an adaptative physiological response may have allowed the E + heifers to maintain a growth rate similar to that of the E− heifers until the final week of the experiment. The reduction in performance observed in the E + heifers during the final week may have been exacerbated by a severe heat wave coupled with high relative humidity (Fig. S1), potentially intensifying the negative effects of fescue toxicosis.

Over the 49-day experimental period, E− heifers gained an average of 59 kg (1.29 kg/day), while E + heifers gained only 44 kg (0.92 kg/day). The reduced ADG, lower BW, and the magnitude of differences between E + and E- heifers observed in this study are consistent with previous research evaluating beef cows and steers grazing endophyte-infected compared to low- or non-toxic pastures. Lucas et al. (2021) reported that cows grazing toxic fescue gained approximately 0.24 kg/day less than cows grazing non-toxic pastures. Similarly, Mote et al. (2020) observed significantly lower ADG (0.5 kg/day) and cumulative weight gains (13.9 kg) in Angus steers grazing toxic fescue compared to those grazing non-toxic tall fescue (0.9 kg/day and 23.2 kg, respectively). Additionally, Rice et al. (1997) showed a reduction in ADG associated with exposure to the fescue toxin, reporting gains of 0.22 kg/day in E + compared to 1.04 kg/day in E− animals.

**Fig. 2.**
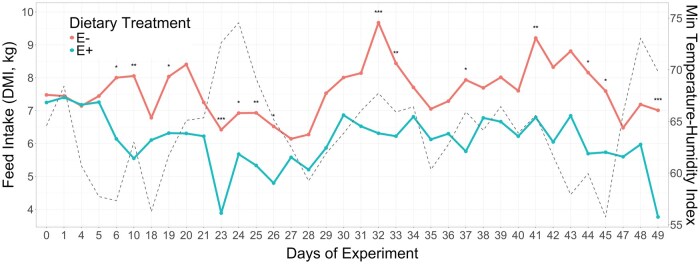
Average daily feed intake of heifers fed endophyte-infected (E+) or endophyte-free (E−) tall fescue seeds, along with the daily minimum temperature-humidity index (dashed line) throughout the experimental period. Asterisks indicate statistical significance between treatments at each time point: *P ≤0.05; **P ≤0.01; ***P ≤0.001. Figure was generated using R package "ggplot2" (https://cran.r-project.org/web/packages/ggplot2/).

Average daily DMI throughout the experiment was significantly affected by fescue toxin ingestion (*P <*0.05, Table 2). Heifers in the E + group consumed less feed when compared with those in the E− group. Figure 2 illustrates the daily variation in DMI for each dietary group over 35 days with accurate feed intake data. A significant difference between the two groups was first detected on day 6 (*P <*0.05) when E + heifers consumed notably less feed (−1.86 ± 0.70 kg of DMI) than E− heifers. DMI of E + heifers never returned to their initial intake levels measured at the start of the trial. Notably, differences in ADG between groups emerged after the second week of exposure (*P <*0.05), even when DMI was included as covariate in the model, suggesting that observed reduction in ADG was not solely attributable to decreased feed intake.

**Table 2.** Least squares mean for feed intake and feeding behavior traits in heifers consuming endophyte-infected (E+) or endophyte-free (E−) tall fescue, with corresponding significance levels.

Traits	E−	E+	SEM	*P*-value
**Average DMI, kg**	8.06	6.95	0.29	0.015*
**Feeding frequency, visits/d**	19.30	18.73	1.16	0.753
**Feeding duration, min/d**	64.4	61.0	5.42	0.663
**Feeding rate, g/min**	220	183	90	0.010**
**Meal frequency, meal/d**	15.80	15.49	1.05	0.758
**Meal duration, min/meal**	4.06	3.99	0.28	0.853
**Meal intake, g/meal**	478	397	20	0.013*

DMI = dry matter intake; Asterisks indicate statistical significance between treatments:

* *P ≤*0.05 and

** *P ≤*0.01.

Additionally, the use of the automated feeding system provided precise and continuous monitoring of individual feeding behavior, enabling reliable comparisons between groups throughout the study period. We found that feeding rate and meal intake differed significantly between the dietary treatments (*P <*0.05; Table 2). E + heifers exhibited a lower feeding rate and smaller meal intake compared with E− heifers. In contrast, feeding frequency, feeding duration, meal frequency, and meal duration were not influenced by the ingestion of the fescue toxin (*P >*0.663). These results suggest that while E + heifers approached the feed bunk and spent similar amounts of time eating as E− heifers, they ate at a slower rate, leading to reduced feed consumption per meal. Because most studies on fescue toxicosis in beef cattle have been conducted under pasture conditions, relatively few have directly documented reductions in feed intake (Osborn et al. 1992; Aldrich et al. 1993). To our knowledge, this is the first study to report differences in feeding behavior associated with the effects of ergot alkaloids in beef cattle.

A possible explanation for the reduced DMI observed in our study is that fescue toxicosis disrupts appetite-regulatory mechanisms through metabolic disturbances including altered energy metabolism, impaired nutrient absorption, reduced digestive efficiency, and hormonal imbalances (Bhusari et al. 2007; Brown et al. 2009; Sarich et al. 2022), using an in vitro rumen simulation technique, demonstrated that ergot alkaloids negatively affect ruminal fermentation and microbial population, leading to reduced feed digestibility. Lower digestibility results in decreased energy availability per unit of feed, which can suppress voluntary intake as animals attempt to conserve metabolic resources (Klotz, 2015). In addition, feeding reluctance and appetite suppression may be further compounded by disturbances in serotonergic and dopaminergic signaling pathways, which regulate appetite and satiety, and prolactin synthesis, respectively (Klotz et al. 2013; Valente et al. 2020).

Interestingly, two heatwave events occurred during our trial, marked by minimum THI values approaching 75 on days 23 − 24 and 48 − 49 (Fig. 2). Among the three THI metrics evaluated, minimum THI showed the strongest negative correlation with DMI (*r = *−0.20), compared to mean (*r = *−0.12) and maximum (*r = *−0.07) THI. This finding is biologically meaningful, as minimum THI likely reflects the nighttime thermal conditions when cattle typically dissipate accumulated heat. If nighttime THI remains elevated, animals may struggle to cool down adequately, limiting their ability to cope with subsequent daytime heat stress. As a result, elevated minimum THI may impair thermoregulatory recovery and contribute to reduced feed intake.

In fact, the two heatwave events coincided with notable reductions in DMI across both dietary groups. To cope with heat stress, animals reduce feed intake as a means of lowering metabolic heat production and minimizing additional heat gain (Dahl et al. 2020). However, the heat waves appeared to exacerbate the negative effects of fescue toxicosis, as DMI was more severely suppressed in E + heifers compared to their E− counterparts. In cattle, THI values above 74 are typically considered indicative of heat stress onset, while values exceeding 80 are associated with severe heat stress and can lead to significant performance losses (Lucas et al. 2021). During the two heatwave periods in our study, average THI values reached 80.5 and 78.9, with maximum THIs of 86.6 and 84.9, respectively (Fig. S1).

### Alterations in thermoregulation induced by fescue toxicosis

**Fig. 3.**
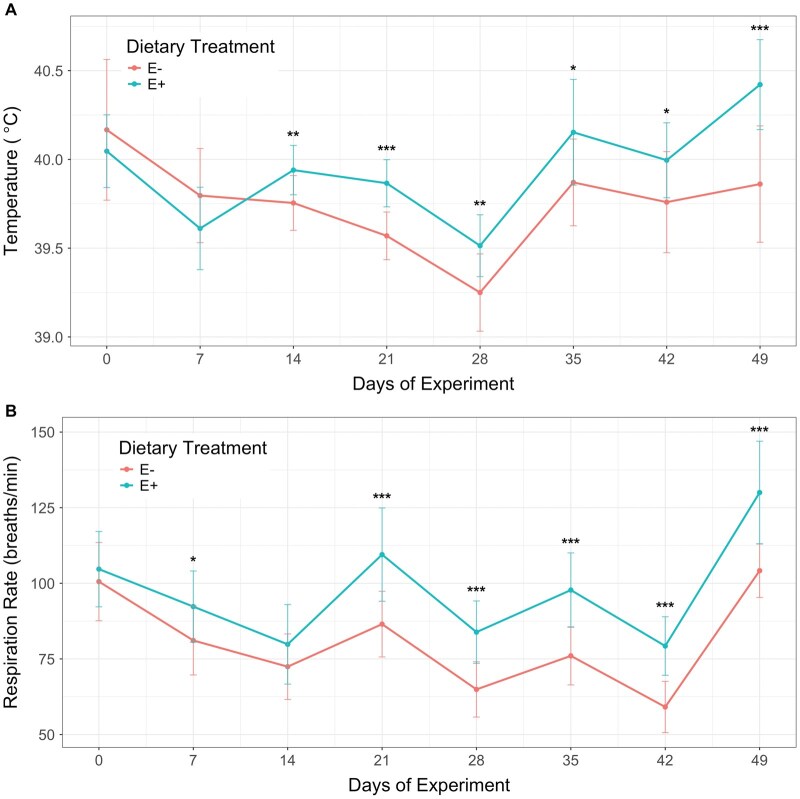
Thermoregulatory responses of heifers fed endophyte-infected (E+) or endophyte-free (E−) tall fescue seeds over a 49-day experimental period. A) Rectal temperature and B) respiration rate were measured weekly. Vertical bars represent the standard deviation for each dietary treatment. Asterisks indicate statistical significance between treatments at each time point: *P ≤0.05; **P ≤0.01; ***P ≤0.001. Figures were generated using R package "ggplot2" ((https://cran.r-project.org/web/packages/ggplot2/)

Rectal temperature in E + heifers began to differ significantly from that of E− heifers after two weeks of exposure to ergot alkaloids (day 14, *P <*0.01), remaining consistently greater for the remainder of the trial (Fig. 3A). Differences in respiration rate emerged even earlier, becoming significant after just one week of exposure (*P <*0.05) and persisting throughout the study (*P <*0.001), except on day 14 (*P = *0.147). Observed fluctuations in rectal temperature and respiration rate were likely influenced by ambient environmental conditions, including temperature and humidity (Supplementary Figure S1). Notably, the two highest peaks in respiration rate (on days 21 and 49) occurred during documented heatwave events, affecting both dietary groups. However, E + heifers appeared more severely impacted than E− heifers, particularly during the second heatwave on day 49. These findings support the use of respiration rate and rectal temperature as potential physiological indicators of fescue toxicosis; however, their interpretation must account for environmental temperature and humidity, which can act as confounding factors. Including a control group in our study was critical to disentangling the effects of heat stress from those of ergot alkaloid exposure.

Supporting our findings, Lucas et al. (2021) reported altered systolic blood pressure and elevated respiration rates in beef cows exposed to toxic fescue during summer months. Similarly, in Angus steers grazing endophyte-infected fescue pastures, increased respiration rates have been documented (Rice et al. 1997; Shoup et al. 2016), and elevated rectal temperatures under heat stress conditions have been observed (Spiers et al. 2004).Toxic ergot alkaloids have been associated with vasoconstriction, which reduces blood flow to peripheral capillary beds and impairs the animal’s ability to dissipate heat, thereby increasing susceptibility to heat stress and elevating respiration rate (Finch 1986; Aldrich et al. 1993). This compromised thermoregulation can further suppress voluntary feed intake as a physiological strategy to minimize internal heat production (Dahl et al. 2020), consistent with the reductions in intake observed in this study. During the heat waves, the core body temperature of E + animals was already elevated and their natural cooling mechanisms were impaired. Consequently, the combined effects of ergot alkaloid toxicity and environmental heat stress acted synergistically, resulting in a sharper and more pronounced decline in feed intake than either factor alone.

### Liver function in response to fescue exposure

Ergot alkaloids are known to exert hepatotoxic effects, potentially impairing liver function by altering hepatic metabolism (Brown et al. 2009). The authors reported increased hepatic metabolic activity and smaller whole liver weights in steers grazing endophyte-infected tall fescue forages. Additionally, hepatic metabolism can be influenced by DMI; variations in DMI can alter liver blood flow and metabolic activity, thereby affecting liver function (Reynolds et al. 2003). Given that DMI differed significantly between the dietary groups in our study (Table 2), we included DMI as a covariate in the statistical model when evaluating the effects of fescue toxicosis on the liver profile traits. This approach allowed us to account for the confounding effects of feed intake level, enabling a more accurate evaluation of the specific impact of ergot alkaloid exposure on liver function. Notably, DMI was significantly negatively associated with AST (−1.306 U/L, *P = *0.043) and showed a tendency toward a positive association with albumin levels (0.057 g/dL, *P = *0.057).

We found that ALP levels were significantly lower in E + heifers compared with E− heifers (*P <*0.05, Table 3), while AST concentrations were greater in the E + group (*P <*0.01). Reduced serum ALP has been previously reported as a biomarker of ergot alkaloid exposure in cattle (Rice et al. 1997; Brown et al. 2009; Jackson et al. 2015). In contrast to those studies, which also observed decreased AST levels, our results align more closely with findings from Rahimabadi et al. (2022), who reported elevated AST concentrations in cattle exposed to ergot alkaloids. While serum enzyme alterations are frequently associated with fescue toxicosis, their underlying physiological causes and implications remain poorly understood.

**Table 3.** Comparison of liver profile traits (least squares means) in heifers fed endophyte-infected (E+) or endophyte-free (E−) tall fescue and associated significance levels.

Traits	E−	E+	SEM	*P*-value
**Albumin, g/dL**	3.71	3.77	0.07	0.420
**Alkaline phosphatase (ALP), U/L**	135.0	101.0	9.88	0.022*
**Aspartate aminotransferase (AST), U/L**	51.2	57.7	1.44	0.005**
**Gama-glutamyltransferase, U/L**	19.3	18.5	0.95	0.567
**Glutamate dehydrogenase, U/L**	15.0	12.8	1.72	0.396
**Cholesterol, mg/dL**	129.0	95.0	8.08	0.008**
**Bilirubin, mg/dL**	0.18	0.16	0.01	0.248
**Triglycerides, mg/dL**	27.4	22.4	2.99	0.255

Asterisks indicate statistical significance between treatments:

* *P ≤*0.05 and

** *P ≤*0.01. The average dry matter intake (DMI) from the five days prior to blood collection was included in the model as a covariate to account for variation in feed intake. DMI was significantly negatively associated with AST (−1.306 U/L, *P = *0.043) and tended toward a positive association with albumin levels (0.057 g/dL, *P = *0.057).

Decreased serum ALP concentrations may reflect reduced digestive efficiency (Schultze et al. 1999). In addition, ALP plays a critical role in facilitating fatty acid absorption in the small intestine (Hansen et al. 2007). Therefore, the reduction in ALP may impair digestive processes and nutrient absorption, potentially contributing to the decreased DMI observed in our study. Elevated AST levels in E + heifers may result from multiple mechanisms, including hepatocellular injury caused by ergot alkaloid metabolism, systemic vasoconstriction that reduces hepatic blood flow and promotes enzyme leakage, and increased metabolic strain due to impaired thermoregulation under high THI conditions (Panteghini 1990; Cui et al. 2016; Rahimabadi et al. 2022). Reduced ALP and elevated AST together reflect metabolic and hepatic stress, emphasizing the compounded impact of fescue toxicosis under concurrent heat stress.

Cholesterol levels were significantly lower in E + heifers compared with E− heifers (*P <*0.01). This reduction may be attributed to alterations in lipid metabolism induced by ergot alkaloid exposure(Stuedemann et al. 1985; Realini et al. 2005). Elevated body temperature, which was observed in E + heifers, may also contribute to decreased serum cholesterol (Blond et al. 2024). Jackson et al. (2015) suggested that cattle exposed to ergot alkaloids may utilize circulating cholesterol to support altered energy demands under toxin-induced stress. The lower cholesterol concentrations observed in our E + heifers are consistent with previous findings in cattle consuming endophyte-infected tall fescue (Bond et al. 1984; Stuedemann et al. 1985; Rice et al. 1997; Oliver et al. 2000; Nihsen et al. 2004).

No significant differences were observed between groups (E + and E−) for GGT, GLDH, albumin, bilirubin, or triglycerides (*P >*0.05). This lack of difference may reflect the animals’ ability to partially compensate for the metabolic disruptions induced by ergot alkaloid exposure. Alternatively, the duration of the trial may not have been sufficient to reveal changes in these parameters, which may require chronic or prolonged exposure to become evident. Jackson et al. (2015) reported an alteration in serum triglyceride, albumin, and bilirubin concentrations in Angus-cross steers under a summer-long grazing of high-endophyte-infected tall fescue. Similar to our findings, Brown et al. (2009) reported no significant differences in GLDH and triglyceride levels in steers grazing endophyte-infected fescue, and Oliver et al. (2000) observed no significant alterations in GGT levels in cattle grazing E + pastures over a three-period, further supporting the notion that some aspects of liver function may be resilient to moderate durations of ergot alkaloid challenge.

Overall, the combination of lower cholesterol, elevated AST, and reduced ALP suggests a metabolic disruption in E + heifers, driven by fescue toxicosis. Although liver function response was assessed at a single time point, these results enhance our understanding of fescue toxicosis in beef cattle and highlight the need for further research into its metabolic consequences.

## Conclusion

Exposure to ergot alkaloids from endophyte-infected tall fescue induces complex and systemic physiological responses in beef heifers, beginning as early as two weeks after exposure. Significant reductions in average daily gain, feed intake, and feeding rate, along with elevated respiration rate and rectal temperature, indicate a rapid onset of metabolic and thermoregulatory dysfunction. A subsequent heat wave further exacerbated the negative impacts in E + heifers, compounding the effects on growth efficiency and physiological stress responses. Elevated AST levels alongside reductions in ALP and cholesterol highlight potential disruptions in liver function and lipid metabolism. Collectively, these results underscore the multifaceted burden imposed by fescue toxicosis on cattle health, thermoregulation, productivity, and metabolic resilience.

We acknowledge that this study involved a limited sample size and that liver function traits were measured only at the end of the experiment; therefore, further research is needed to deepen our understanding of the systemic impacts of ergot alkaloid exposure.

## Supplementary Material

txaf171_Supplementary_Data

## List of abbreviations:

ADG: average daily gain
ALB: albumin
ALP: alkaline phosphatase
AST: aspartate aminotransferase
BW: body weight
DMI: dry matter intake
GGT: gamma-glutamyl transferase
GLDH: glutamate dehydrogenase
RH—: relative humidity
THI: temperature-humidity index
