# Supplementary material for: Performance, thermoregulation, and liver function in beef heifers exposed to endophyte-infected or endophyte-free tall fescue under a common environment
Source: Transl Anim Sci. 2026 Jan 9;10:txaf171. doi: 10.1093/tas/txaf171 (PMC12908664; doi:10.1093/tas/txaf171)
Supplement: txaf171_Supplementary_Data [file txaf171_supplementary_data.zip › SupplementaryMaterial.docx]

**Supplementary Material**

**Table S1.** Experimental design and animal assignment details, including heifer ID, corresponding dam and sire, pen allocation, and dietary treatment (endophyte-infected [E+] or endophyte-free [E−] tall fescue seed).

| Heifer ID | Dam ID | Sire ID | Pen | Dietary Treatment |
| --- | --- | --- | --- | --- |
| 163K | 120H | High Life | 1 | E+ |
| 233K | 500H | Rapid Fire | 1 | E+ |
| 255K | 607H | Rest Easy | 1 | E+ |
| 160K | 469H | Standout | 1 | E+ |
| 140K | 271H | High Life | 1 | E+ |
| 175K | 360H | Rapid Fire | 1 | E+ |
| 164K | 316H | High Life | 2 | E+ |
| 128K | 420H | Rapid Fire | 2 | E+ |
| 154K | 129H | Rapid Fire | 2 | E+ |
| 250K | 553H | Rest Easy | 2 | E+ |
| 196K | 324H | Standout | 2 | E+ |
| 273K | 522H | Standout | 2 | E+ |
| 143K | 231H | Standout | 3 | E− |
| 157K | 170H | Rest Easy | 3 | E− |
| 171K | 503H | Rapid Fire | 3 | E− |
| 155K | 151H | High Life | 3 | E− |
| 177K | 516H | High Life | 3 | E− |
| 257K | 135H | Rapid Fire | 3 | E− |
| 165K | 177H | High Life | 4 | E− |
| 133K | 166H | Rapid Fire | 4 | E− |
| 166K | 537H | Rapid Fire | 4 | E− |
| 126K | 293H | Rest Easy | 4 | E− |
| 176K | 412H | Standout | 4 | E− |
| 206K | 270H | Standout | 4 | E− |

**Table S2.** Summary of blood biochemical markers assessed in this study, including their tissue of origin, biological classification, primary location, physiological function, and literature references. These markers reflect hepatic function, metabolic activity, and systemic responses relevant to the evaluation of fescue toxicosis in beef heifers.

| Origin | Type | Location | Function | References |
| --- | --- | --- | --- | --- |
| Hepatocellular | Albumin (ALB) | Serum | Maintains osmotic pressure and transport | 1 |
|  | Alkaline Phosphatase (ALP) | Cell membranes in the liver, bone, kidney, and intestine | Hydrolysis of phosphate esters; important in bone formation and fat metabolism | 1 |
|  | Aspartate Aminotransferase | Mitochondrial and cytosolic in liver and cardiac cells | Amino acid catabolism and krebs cycle | 1 |
|  | Gamma-glutamyltransferase (GGT) | Cytosolic in renal tubular and hepatic cells | Transferase activity in amino acid transport and metabolism | 2 |
|  | Glutamate Dehydrogenase (GHDL) | Mitochondrial in liver cells | Catabolic enzyme in the TCA cycle | 2 |
|  | Bilirubin (BIL) | Mostly in liver, lesser in spleen and kidneys | Breakdown product of heme, excreted in bile | 1 |
| Plasma | Cholesterol (COL) | Cell membrane lipid layers | Component of cell membranes and precursors to steroid hormones | 3 |
|  | Triglycerides (TRI) | Stored in fat cells, circulate in blood bound to lipoproteins | Energy storage and metabolism | 3 |

^1^Gelain, M.E. (2022), Veterinary hematology, clinical chemistry, and cytology Mary Ann Thrall, Glade Weiser, Robin W. Allison, and Terry W. Campbell, Wiley Blackwell, ISBN978-1-119-28640-0, 1042 pages, 2022. Vet Clin Pathol, 51: 612-613.

^2^Watson, J. L., J. A. Angelos, K. A. Clothier, K. E. Estell, J. E. Madigan, B. P. Smith, S. J. Spier, B. A. Sponseller, J. W. Norris, and F. Tabin. 2020. Diseases of the Hematopoietic and Hemolymphatic Systems. In: Large Animal Internal Medicine. Elsevier. p. 1151-1196.e11.

^3^Smith, G. W., J. L. Davis, J. B. Malone, T. G. Nagaraja, J. E. Tomlinson, and V. Dore. 2020. Diseases of the Hepatobiliary System. In: Large Animal Internal Medicine. Elsevier. p. 921-955.e6.

**Table S3.** Least squares mean for body weight (BW,), average daily gain (ADG), rectal temperature (RT), and respiration rate (RR) of heifers consuming endophyte-infected (E+) or endophyte-free (E−) tall fescue seeds over a 49-day experimental period, with corresponding significance levels.

| Item | E− | E+ | SEM | P-value |
| --- | --- | --- | --- | --- |
| BW, kg |  |  |  |  |
| day 0 | 369 | 366 | 10.00 | 0.820 |
| day 7 | 376 | 374 | 1.61 | 0.444 |
| day 14 | 387 | 374 | 1.71 | <0.001*** |
| day 21 | 393 | 383 | 2.64 | 0.013* |
| day 28 | 401 | 391 | 2.66 | 0.013* |
| day 35 | 412 | 398 | 2.97 | 0.004** |
| day 42 | 421 | 411 | 3.35 | 0.034* |
| day 49 | 428 | 410 | 3.62 | 0.002** |
| ADG, kg |  |  |  |  |
| day 0-7 | 1.17 | 0.90 | 0.23 | 0.417 |
| day 0-7^§^ | 1.04 | 1.02 | 0.23 | 0.950 |
| day 7-14 | 1.69 | 0.06 | 0.28 | <0.001*** |
| day 7-14^§^ | 1.45 | 0.30 | 0.29 | 0.019* |
| day 14-21 | 0.78 | 1.19 | 0.26 | 0.283 |
| day 14-21^§^ | 0.48 | 1.49 | 0.25 | 0.014* |
| day 21-28 | 1.17 | 1.17 | 0.27 | 0.988 |
| day 21-28^§^ | 1.07 | 1.27 | 0.30 | 0.653 |
| day 28-35 | 1.52 | 1.01 | 0.23 | 0.129 |
| day 28-35^§^ | 1.47 | 1.06 | 0.23 | 0.226 |
| day 35-42 | 1.39 | 1.81 | 0.26 | 0.263 |
| day 35-42^§^ | 1.36 | 1.83 | 0.27 | 0.230 |
| day 42-49 | 0.99 | -0.03 | 0.19 | 0.001*** |
| day 42-49^§^ | 0.99 | -0.03 | 0.16 | <0.001*** |
| RT, ^o^C |  |  |  |  |
| day 0 | 40.17 | 40.05 | 0.09 | 0.360 |
| day 7 | 39.80 | 39.61 | 0.07 | 0.082 |
| day 14 | 39.75 | 39.94 | 0.042 | 0.005** |
| day 21 | 39.57 | 39.87 | 0.04 | <0.001*** |
| day 28 | 39.25 | 39.51 | 0.06 | 0.003** |
| day 35 | 39.87 | 40.15 | 0.08 | 0.019* |
| day 42 | 39.76 | 40.00 | 0.07 | 0.031* |
| day 49 | 39.86 | 40.42 | 0.08 | <0.001*** |
| RR, breaths/min |  |  |  |  |
| day 0 | 101.0 | 105.0 | 3.67 | 0.434 |
| day 7 | 81.1 | 92.3 | 3.35 | 0.027* |
| day 14 | 72.4 | 79.8 | 3.48 | 0.147 |
| day 21 | 86.5 | 109.5 | 3.85 | <0.001*** |
| day 28 | 64.9 | 83.9 | 2.81 | <0.001*** |
| day 35 | 76.0 | 97.8 | 3.18 | <0.001*** |
| day 42 | 59.1 | 79.3 | 2.63 | <0.001*** |
| day 49 | 104.0 | 130.0 | 3.91 | <0.001*** |

^§^ADG model with weekly average dry matter intake as a covariate to determine whether the effect of E+ ingestion on ADG was primarily driven by differences in feed intake. Asterisks indicate statistical significance between treatments at each time point: *P ≤ 0.05; **P ≤ 0.01; ***P ≤ 0.001.

**
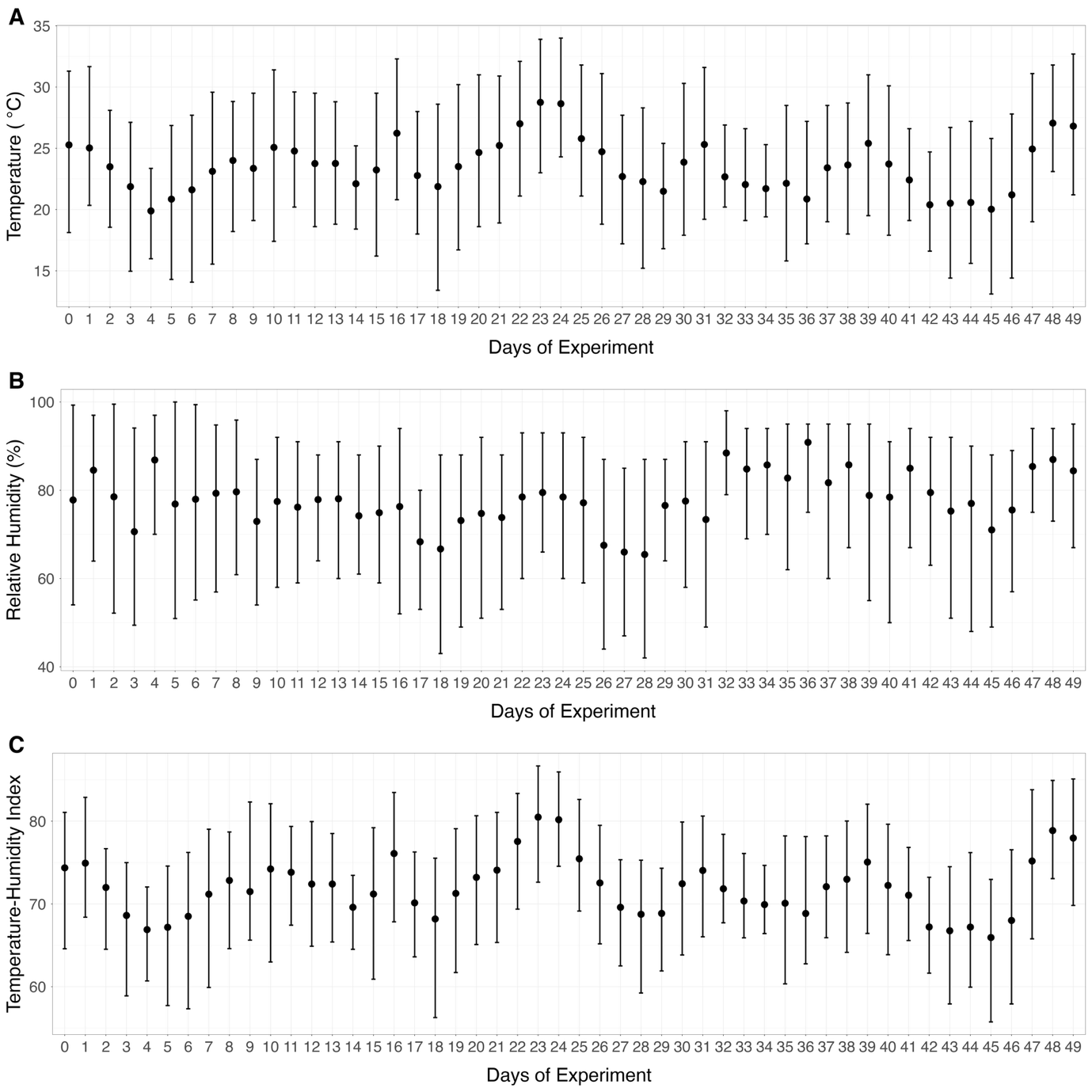
**

**Figure S1.** Environmental conditions during the 49-day experimental period. Dots represent daily mean values, while vertical bars indicate the corresponding minimum and maximum values. Temperature–humidity index (THI) thresholds of 68–72 indicate mild heat stress, and values exceeding 80 indicate severe heat stress.
